# HBV-associated intrahepatic cholangiocarcinoma with high serum alpha-fetoprotein: a case report with review of literature

**DOI:** 10.1186/s12879-016-1643-7

**Published:** 2016-06-14

**Authors:** Caixia Wang, Haiyan Jing, Dan Sha, Weibo Wang, Jianpeng Chen, Yangang Cui, Junqing Han

**Affiliations:** Department of Medical Oncology, Shandong Provincial Hospital Affiliated to Shandong University, 324 Jing Wu Road, Jinan, Shandong Province 250021 People’s Republic of China; Department of Pathology, Shandong Provincial Hospital Affiliated to Shandong University, 324 Jing Wu Road, Jinan, Shandong Province 250021 People’s Republic of China; Department of Radiotherapy, Shandong Provincial Hospital Affiliated to Shandong University, 324 Jing Wu Road, Jinan, Shandong Province 250021 People’s Republic of China

**Keywords:** Intrahepatic cholangiocarcinoma, HBV infection, AFP, Antiviral therapy

## Abstract

**Background:**

Intrahepatic cholangiocarcinoma (ICC) is a rare malignant tumor. The etiology of ICC remains poorly understood. Recently, hepatitis B virus (HBV) infection has been implicated as a potential risk factor for ICC, particularly in HBV-endemic areas. Elevation of serum alpha-fetoprotein (AFP) is seen in approximately 20 % of ICC patients. However, serum AFP levels higher than 10,000 ng/mL have only been reported in a few ICC patients. We report an unusual case of HBV-associated ICC occurring in a male with a markedly elevated serum AFP.

**Case presentation:**

A 60-year-old East Asian male presented with complaints of epigastric distention and right shoulder pain. Laboratory tests showed HBV infection, HBV deoxyribonucleic acid (DNA) slightly elevated (21 IU/mL) and serum AFP markedly elevated (12,310 ng/mL). Computed tomography (CT) scan found a large and irregular mass in the left lobe of the liver. The patient underwent the left hepatic lobe resection. Histopathological examination showed chronic hepatitis B in the background liver and the immunohistochemical (IHC) findings strongly supported the diagnosis of ICC with aberrant expression of AFP. Serum AFP and HBV DNA declined to normal level postoperatively. The patient received four cycles of gemcitabine plus oxaliplatin and took entecavir to prevent HBV reactivation. The patient kept disease free for 18 months in the latest follow-up.

**Conclusion:**

ICC patients with HBV infection should be distinguished from other ICC cases, based on distinct clinicopathological features and favorable outcome. Screening for HBV infection should be carried out before initiation of chemotherapy. Antiviral therapy is indicated for prevention of HBV reactivation.

## Background

ICC is a rare malignant tumor arising from peripheral intrahepatic biliary epithelia. It accounts for 5 % of all primary liver malignancies, second only to hepatocellular carcinoma (HCC) [1]. ICC is often an incidental radiologic finding. Thus, clinical presentation alone is non-specific and insufficient for diagnosis. The diagnosis of ICC should be viewed as a diagnosis of exclusion. Due to the aggressive nature of ICC, most patients are diagnosed with lymph node involvement, intrahepatic metastasis, and peritoneal dissemination. ICC is refractory to chemotherapy and radiotherapy, and an R0 resection remains the only potentially curative option [2]. Unfortunately, advanced ICC patients have dismal prognosis with a median survival of less than 24 months [3]. Although several potential risk factors driving genetic heterogeneity in cholangiocarcinoma have been reported, such as liver fluke infection, primary sclerosing cholangitis, hepatolithiasis and asbestos, the etiology of ICC remains poorly understood [4, 5]. Recently, HBV infection—one of the major causes of HCC—has also been implicated as a potential risk factor for ICC, particularly in HBV-endemic areas [6]. AFP—primarily synthesized in the liver and yolk sac of developing embryo—is also a widely used tumor marker of HCC or germ cell tumor. Elevation of serum AFP (higher than 20 ng/mL) is seen in approximately 20 % of ICC patients [1]. However, serum AFP levels higher than 10,000 ng/mL have only been reported in a few ICC patients. We report an unusual case of HBV-associated ICC occurring in a 60-year-old male with a markedly elevated serum AFP. Written informed consent was obtained from the patient.

## Case presentation

### Clinical presentation

A 60-year-old East Asian male was admitted to the Shandong Provincial Hospital Affiliated to Shandong University (Jinan, China) in July 2014, with complaints of epigastric distention and right shoulder pain for the past six months. His past medical history included prolapse of a lumbar intervertebral disc and hypertension, both of which were under control. Family history indicated the patient’s mother was a hepatitis B patient. Physical examination revealed tenderness over the epigastric and right subcostal areas.

### Lab findings

Lab tests showed hepatitis B surface antigen (HBsAg), hepatitis B e antibody (HBeAb) and hepatitis B core antibody (HBcAb) were positive, while hepatitis B e antigen (HBeAg) and hepatitis B surface antibody (HBsAb) were negative. The serum HBV DNA level was slightly elevated to 21 IU/mL (normal level:< 20 IU/mL), measured by highly sensitive HBV DNA detection tools. Hepatitis C virus (HCV) antibody was negative. Gamma glutamyltransferase (GGT) was mildly elevated to 69 U/L (normal level: 10 U/L - 60 U/L). Other liver function tests, including total bilirubin, conjugated bilirubin, alkaline phosphatase, alanine aminotransferase (ALT), aspartate aminotransferase (AST), total protein and albumin, were all within normal limits. Of the tumor markers tested, AFP was 12,310 ng/mL (normal level: 0 ng/mL −20 ng/mL). Carcinoembryonic antigen (CEA), carbohydrate antigen 19–9 (CA19-9) and carbohydrate antigen 125 (CA125) were within normal limits.

### Image findings

**Fig. 1:**
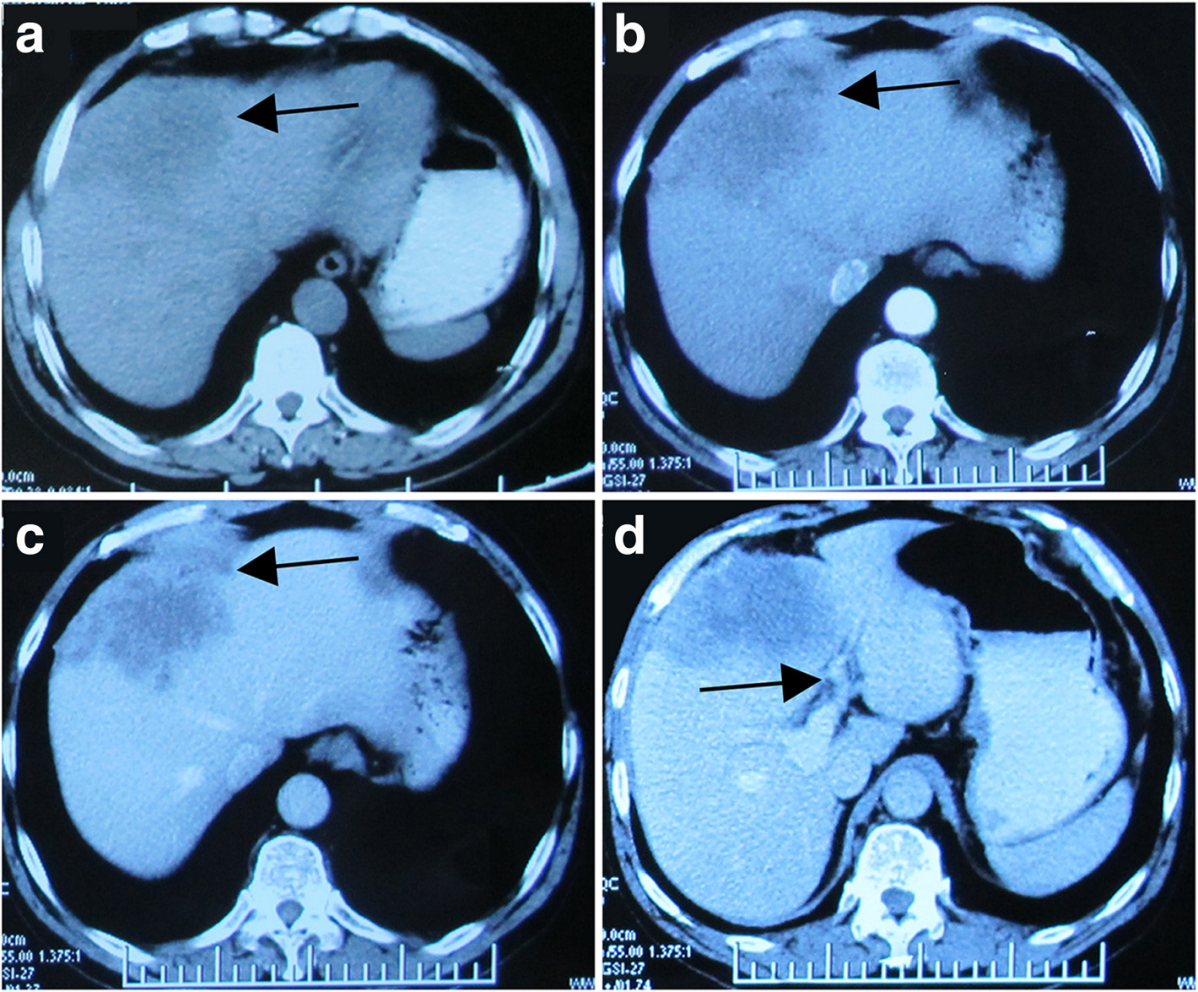
A 60-year-old man with epigastric distention and right shoulder pain. The axial CT scan showed a large (10.2 cm × 8.6 cm × 6.3 cm), lobulated and irregular mass in the left hepatic lobe (segment IV). **a** Pre-contrast CT scan: the ill-defined heterogeneous hypodense lesion (*arrows*). **b** Contrast-enhanced CT scan (arterial phase): the hypoattenuated mass with irregular margin lesion was not enhanced on the arterial phase. A dilatation of intrahepatic bile ducts (*arrow*). **c** Contrast-enhanced CT scan (portal phase): delayed vague enhancement in the portal phase, indicating intrahepatic cholangiocarcinoma (ICC). Minimal peripheral enhancement observed during the phases (*arrow*). **d** Contrast-enhanced CT scan (portal phase): a low-attenuation lesion in the left side of the portal vein (*arrow*). Note the mild infiltration adjacent to the tumor

The axial CT scan showed a large (10.2 cm × 8.6 cm × 6.3 cm), lobulated and irregular mass in the left lobe of the liver (segment IV), which was not enhanced on the arterial phase but only vaguely enhanced on the portal phase, suggesting intrahepatic cholangiocarcinoma (ICC) (Fig. 1).

### Surgery

The patient was admitted to surgery on July 22, 2014. Intraoperatively, significant nodular cirrhosis was noted and a hard tumor with a diameter of about 11 cm was palpable in the left hepatic lobe. It invaded the liver capsule and gallbladder bed. No lymphadenopathy or ascites was found. No tumor implantation or distant metastases were found in the omentum or pelvic cavity. The patient underwent left hepatic lobe resection and cholecystectomy.

### Pathological findings

**Fig. 2:**
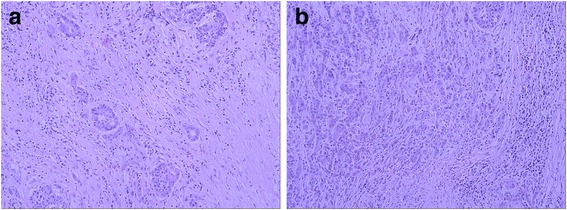
Histology of cholangiocarcinoma with stem cell features. **a** Cholangiocarcinoma composed of duct-like structures lined by cuboidal or columnar cells with high pleomorphism, surrounded by dense stromal reaction. H&E 200×. **b** Focal tumor cells showed stem cell-like liver cancer features. H&E 200×

**Fig. 3:**
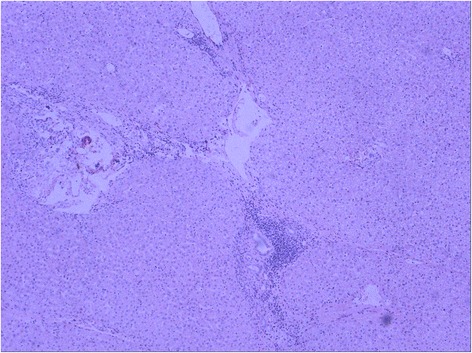
Histology of non-tumor parenchyma showing chronic hepatitis B. H&E 100×

**Fig. 4:**
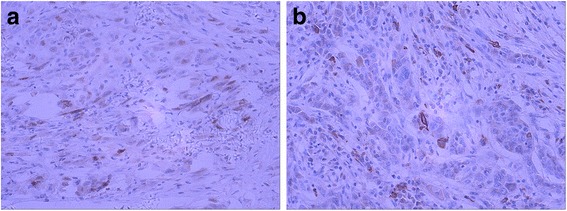
High-power photomicrograph showing immunoreactivity of cholangiocarcinoma. Focal tumor cells positive for two liver stem-cell markers, P63 (**a**) and CK14 (**b**). H&E 400×

Histopathologic examination showed a nonencapsulated, moderately or poorly differentiated adenocarcinoma. The tumor cells were arranged in duct-like structures against a background of broad desmoplastic stroma (Fig. 2a). The tumor cells were cuboidal to columnar in shape with round and medium-sized nuclei, prominent nucleoli, frequent mitotic activity, and a moderate amount of eosinophilic cytoplasm. Some tumor cells appeared undifferentiated with stem cell-like features (Fig. 2b), with no definite histological features of hepatocytes and cholangiocytes. Massive coagulative tumor necrosis involved approximately 40 % of the tumor volume. Extensive sampling of the tumor failed to demonstrate histologic evidence of hepatoid differentiation. No histologic features of yolk-sac tumor were recognized. The background liver showed chronic hepatitis B (Fig. 3). Because the clinical impression and histopathologic findings were inconsistent, we conducted IHC studies with a panel of antibodies. The results showed that the tumor cells were strongly and diffusely positive for AFP, whereas the adjacent non-neoplastic hepatocytes and bile ducts were completely negative. The tumor cells were also strongly and diffusely positive for cytokeratins CK7 and CK19 with a luminal staining pattern. However, the tumor cells were completely negative for hepatocyte antigen, Glypican-3, CK20, and CDX-2. The antibodies to liver stem cells/progenitor cells, including KIT (CD117), CD34, CD56, cytokeratin CK14 and P63, were used to explore the possibility of stem cell origin. Immunostaining of P63 and CK14 was positive in small cells within the “stem cell-like” zone (Fig. 4). These IHC findings strongly supported the diagnosis of ICC with aberrant expression of AFP.

### Chemotherapy and antiviral therapy

At postoperative weeks 1, 5 and 8, the patient’s AFP level declined to 628.4 ng/mL, 43 ng/mL and 15.7 ng/mL, respectively (normal level: 0 ng/mL −20 ng/mL). From August 30, 2014 to November 10, 2014, the patient received gemcitabine (1800 mg on days 1 and 8) plus oxaliplatin (200 mg on day 1) every 3 weeks for four cycles. The patient tolerated chemotherapy very well with mild gastrointestinal and hematological toxicities. One week before chemotherapy, he started taking entecavir dispersible tablet (0.5 mg po qn), continued during chemotherapy and for 6 months after chemotherapy. The patient was followed up in the outpatient department. The latest follow-up on March 25, 2016, showed no evidence of recurrence. His AFP level was 12.1 ng/mL (normal level: 0 ng/mL −20 ng/mL) and HBV DNA declined to normal level (<20 IU/mL).

## Conclusions

This case report involves a patient diagnosed with HBV-associated ICC and high serum AFP level. Generally speaking, HBV infection is regarded as one of the major causes of HCC. AFP is regarded as a tumor marker of HCC. Compared with HCC, ICC presents a more dismal course, mainly due to frequent lymphatic involvement, periductal invasion, poorly encapsulated tumors, or difficult early diagnosis [7]. CA19-9 is regarded as a tumor marker of ICC. However, recent evidence suggested a possible etiological role for HBV infection in ICC, especially in HBV-endemic areas [6, 8, 9]. HBV-associated ICC and ICC without HBV infection manifest significant differences in clinicopathology and prognosis. Patients with HBV-associated ICC are younger, more likely to have elevated serum AFP levels and lower abnormal serum CA19-9 levels. Morphologically, HBV-associated ICC is more likely to be mass-forming rather than intraductal/periductal type. Pathologically, lymphatic involvement is lower and tumor differentiation is poorer in HBV-associated ICC, with a higher prevalence of capsule formation and liver cirrhosis [8]. Therefore, HBV-associated ICC shows a more favorable prognosis compared with ICC without HBV infection. The 1-, 3- and 5-year overall survival (OS) in ICC patients were 42, 18 and 15 % in the HBV-positive group and 24, 12 and 0 % in the HBV-negative group, respectively (*P* = 0.005) [8]. Further, HBV-associated ICC shares many clinicopathological similarities with HBV-associated HCC, such as nearly identical age and sex distribution profiles. This finding suggests that HBV-associated ICC and HCC may involve common carcinogenic processes and originate from hepatic stem cells that undergo malignant transformation [7, 10].

Adult hepatic stem cells are hepatic oval cells, which potentially differentiate into hepatic and hepatobiliary cells, and AFP is an important marker in hepatic stem cells. A study conducted by the liver cancer study group of Japan showed that 4.9 % (10/205) of all ICC patients had a serum AFP level more than 1000 ng/mL, 1 % (2/205) higher than 10,000 ng/mL, and only 0.5 % (1/205) of the ICC patients had a serum AFP level greater than 100,000 ng/mL [1]. In our case, the patient’s serum AFP level was markedly elevated to 12,310 ng/mL and the tumor cells in the liver were strongly and diffusely positive for AFP. IHC revealed uniform expression of CK7 and CK19 but was negative for hepatocyte antigen and Glypican-3 in tumor cells, which argues against the pathological diagnosis of HCC or combined HCC and cholangiocarcinoma. The tumor was finally diagnosed as an ICC with aberrant expression of AFP. Meanwhile, the cancer cells with stem cell-like features were focally positive for hepatic stem cell markers (P63 and CK14), suggesting origin from hepatic stem cells.

The hypothesis of liver tumors having the common origin from hepatic stem cells can be confirmed by report of combined hepatocellular and cholangiocellular carcinoma, a rare neoplasm with an intermediate biological behavior and prognosis between HCC and ICC [11]. Recent studies also reported a case of AFP-producing ICC (serum AFP level: 1,560 ng/mL) expressing stem cell markers (CK14 and CD133) and AFP-producing cells in cholangiocarcinoma possessing cancer stem cell-like traits [12, 13]. The verification of ICC originating from hepatic stem cells requires additional studies.

To date, the mechanism of HBV-related carcinogenesis in ICC remains unclear. Integration of HBV DNA into the human genome—which alters gene expression, chromosomal instability and modification of genomic methylation status—is one of the most important steps in HCC-related carcinogenesis [14]. The effect of HBV DNA on ICC-related carcinogenesis is unclear. HBV X gene-encoded protein (HBx) acts as a transactivator of various cellular genes mediating HCC-related carcinogenesis [15]. HBx is also present in cancerous and non-cancerous cells in HBV-infected ICC specimens [16]. Further, HBx transfection induces human telomerase reverse transcriptase (hTERT) mRNA expression in cultured normal human cholangiocytes, [17] suggesting that HBx may contribute to the carcinogenesis of biliary epithelia.

Multidisciplinary treatment of ICC includes surgery, locoregional therapy (radiotherapy, TACE and ablation) and systemic therapy (chemotherapy). Complete surgical resection remains the only potentially curative option for patients with ICC. The 5-year survival rate for patients who undergo resection is only 25 to 35 % and most patients suffer from disease recurrence, which highlights the need for more effective systemic therapy [2]. The combination regimen of gemcitabine and cisplatin is an international practice standard, which prolongs median OS compared with gemcitabine alone (11.7 m vs. 8.1 m) [18]. In our case study, the patient received surgery and gemcitabine combined with platinum-based adjuvant chemotherapy, and remained disease-free in the latest follow-up. Anti-cancer therapy leads to complications including HBV reactivation, which ranged from 20 to 70 % in previous reports [19]. The Practice Guidelines of the American Association for the Study of Liver Diseases (AASLD) recommend HBV screening before chemotherapy [20]. Antiviral therapy is indicated for HBsAg-positive/anti-HBc–positive patients before and during chemotherapy, and for approximately 6 to 12 months after completing chemotherapy [21]. In our case study, the patient was HBsAg-positive/anti-HBc–positive and entecavir was used to prevent HBV reactivation.

In conclusion, HBV-associated ICC and HCC may share a common carcinogenic mechanism originating in hepatic stem cells. Patients with HBV infection should be distinguished from other ICC cases, based on distinct clinicopathological features and favorable outcome. Screening for HBV infection should be carried out before initiation of chemotherapy. Antiviral therapy is indicated for prevention of HBV reactivation.

## Abbreviations

AASLD, the American Association for the Study of Liver Diseases; AFP, alpha-fetoprotein; ALT, alanine aminotransferase; AST, aspartate aminotransferase; CA125, carbohydrate antigen 125; CA19-9, carbohydrate antigen 19-9; CEA, carcinoembryonic antigen; CT, computed tomography; DNA, deoxyribonucleic acid; GGT, gamma glutamyltransferase; HBcAb, hepatitis B core antibody; HBeAb, hepatitis B e antibody; HBeAg, hepatitis B e antigen; HBsAb, hepatitis B surface antibody; HBsAg, hepatitis B surface antigen; HBV, hepatitis B virus; HBx, HBV X gene-encoded protein; HCC, hepatocellular carcinoma; HCV, hepatitis C virus; hTERT, human telomerase reverse transcriptase; ICC, intrahepatic cholangiocarcinoma; IHC, immunohistochemical; OS, overall survival.
